# lncRNA PROX1-AS1 mediates the migration and invasion of placental trophoblast cells via the miR-211-5p/caspase-9 axis

**DOI:** 10.1080/21655979.2021.1953213

**Published:** 2021-07-21

**Authors:** Dan Tang, Li Geng, Jing Ma

**Affiliations:** Obstetrics Department, First Affiliated Hospital of Kunming Medical University, Kunming, China

**Keywords:** lncRNA PROX1-AS1, preeclampsia, trophoblast cells, migration, invasion

## Abstract

Preeclampsia (PE) is a potentially fatal pregnancy complication; however, its pathogenesis remains unclear. Long non-coding RNAs (lncRNAs) are associated with occurrence and progression of PE. Therefore, this study aimed to explore the function of the lncRNA prospero homeobox 1-antisense RNA 1 (PROX1-AS1) and elucidate its underlying molecular mechanism of action. We found that the expression levels of PROX1-AS1 were elevated in both the placental tissues and blood samples of the patients with PE. Moreover, the results of the flow cytometry and transwell assay showed that the knockdown of PROX1-AS1 inhibited the apoptosis and promoted the migration and invasion of HTR-8/SVneo cells. We also assessed the interactions between PROX1-AS1, caspase-9, and microRNA (miR)-211-5p via dual-luciferase reporter and RNA pull-down analyses. The data indicated that PROX1-AS1 acted as a sponge for miR-211-5p to regulate the expression of caspase-9. Moreover, the expression of miR-211-5p was reduced in PE and negatively related to PROX1-AS1, while that of caspase-9 was increased in PE and negatively regulated by miR-211-5p. Furthermore, inhibition of miR-211-5p rescued the facilitation of cell apoptosis, migration and invasion induced by the knockdown of PROX1-AS1. We also found that caspase-9 improved the apoptosis rate, and suppressed the cell migration and invasion induced by the overexpression of miR-211-5p. In conclusion, the knockdown of PROX1-AS1 promoted the cell morbidity of the trophoblast cells by modulating the miR-211-5p/caspase-9 axis, which may alleviate the progression of PE. This novel regulatory network may contribute to the pathogenesis and progression of PE.

## Graphical Abstract

**Figure:**
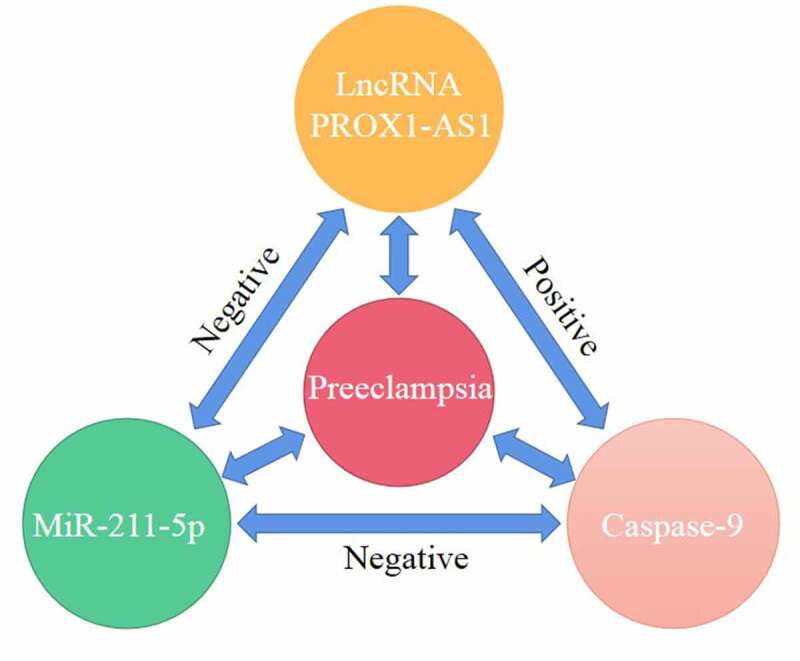

## Introduction

Preeclampsia (PE) is a multisystem disorder of pregnancy that leads to perinatal and fetal death [1]. PE usually presents with hypertension and proteinuria in the third trimester of pregnancy and may lead to the dysfunction of the kidneys, liver, and lungs [2,3]. In 2001, nearly 5% of pregnant women are affected by PE [4]. Hypertensive disorders and inflammation caused by changes in the maternal immune system are the key factors associated with PE [5]. Although the use of aspirin can prevent PE, there is no effective way to predict its occurrence, nor can it be prevented by the prophylactic use of aspirin [6]. Currently, the only way to treat PE is to terminate the pregnancy and deliver the neonate and placenta [3]. Moreover, the pathogenesis of PE has not yet been fully elucidated.

Long non-coding RNAs (lncRNAs) are more than 200 nt in length and they collectively participate in the modification of chromatin and genome structure, maintenance of RNA stability, and transcription [7]. Dysregulated lncRNAs in PE degrade the cellular functions of trophoblast cells, including their immune response, epigenetic regulation, decidualization, and energy metabolism pathways [8–10]. For instance, the lncRNA inhibin beta A (INHBA)-AS1 mediates the proliferation, invasion, and migration of the placental trophoblast cells [11]. Additionally, the knockdown of lncRNA HOXD-AS1 alleviates the progression of PE by enhancing the proliferation of cells and the formation of colonies [12]. Another lncRNA, colon cancer associated transcript 1 (CCAT1), is highly expressed in patients with PE; however, knockdown of CCAT1 increases the cell proliferation and facilitates the cell cycle progression of trophoblastic cells [13]. The lncRNA PROX1-AS1 is upregulated at the early onset of PE [14]; however, the specific role of PROX1-AS1 in the pathogenesis of PE remains unknown.

Therefore, our study aimed to investigate the effects of PROX1-AS1 on the migration and invasion of placental trophoblast cells and elucidate the underlying regulatory molecular mechanisms associated with the pathogenesis of PE. We hypothesized that PROX1-AS1 promotes the cell morbidity of the trophoblast cells by modulating the miR-211-5p/caspase-9 axis

## Materials and methods

### Placenta tissue and blood specimens

Pregnant women diagnosed with PE (n = 40) at the First Affiliated Hospital of Kunming Medical University for our study along with 40 normal pregnant women as healthy controls, all volunteered for this study. All participants provided written informed consent. Clinical specimens were collected from the venous blood samples of the participants. The placenta tissues were collected immediately after placental delivery. Tissues (1 cm × 1 cm × 1 cm) were cut from the umbilical root of the maternal side of the placenta and stored in liquid nitrogen. The clinical information of all participants is shown in Table 1. This study protocol was reviewed and approved by the ethics committee of First Affiliated Hospital of Kunming Medical University.

**Table 1.** Clinical information of patients with PE and healthy control

Factors	Healthy (n = 40)	PE (n = 40)	P-value
Age (years)	28.68 ± 3.25	29.14 ± 3.88	0.5671
Pregnancy BMI	21.03 ± 2.98	21.82 ± 3.11	0.2496
Gestational age (weeks)	38.22 ± 3.17	36.54 ± 2.16	0.0070
Systolic blood pressure (mmHg)	112.52 ± 3.44	168.05 ± 5.02	<0.0001
Diastolic blood pressure (mmHg)	75.26 ± 3.99	108.36 ± 4.54	<0.0001
Proteinuria (g/day)	NA	>0.3	NA
Fetal birth weight (g)	3324.25 ± 305.15	2408.66 ± 397.65	<0.0001

### Cell culture and transfection

Human trophoblast cells (HTR-8/SVneo) were obtained from the American Type Culture Collection (ATCC). They were maintained in the Roswell Park Memorial Institute (RPMI)-1640 medium supplemented with 10% fetal bovine serum (FBS) and 1% penicillin-streptomycin (all from Invitrogen, Carlsbad, CA, USA). The culture conditions included a temperature of 37°C and 5% carbon dioxide (CO_2_).

Small interfering RNA (siRNA)-negative control (nc) and the si-PROX1-AS1 (PROX1-AS1 siRNAs) target the following sequences: PROX1-AS1 siRNA-1, forward: 5′-GCAGCAGAUUUACGGCAAATT-3′ and reverse: 5′-UUUGCCGUAAAUCUGCUGCTT-3′; PROX1-AS1, siRNA-2, forward: 5′-GCCUGGAUAUGUUGUAGUATT-3′ and reverse: 5′-UACUACAACAUAUCCAGGCTT-3′. The empty vectors and caspase-9 overexpression vectors were purchased from Sangon Biotech (Shanghai, China). The miR-211-5p mimic and inhibitor and their corresponding nc were purchased from Invitrogen. Then, the HTR-8/SVneo cells were seeded into 24-well plates and transfected at 37°C with 5% CO_2_ using Lipofectamine 2000 (Invitrogen) until the cell confluency reached 80%.

### Quantitative reverse transcription-polymerase chain reaction (qRT-PCR)

First, total RNA was isolated from the placental tissues, blood, and cells using the TRIzol reagent. After determination of the quality and quantity, equal amount of RNA was reverse transcribed to complementary DNA (cDNA) and qRT-PCR was performed using the GoTaq 1-step RT-qPCR System (Promega, Madison, WI, USA) under the following conditions: ≥ 37°C for 15 min (reverse transcription), 95°C for 10 min (RT inactivation/Hot-star activation), 95°C for 10 s, 40 cycles at 60°C for 30 s, 72°C for 30 s (qPCR), and 60–95°C (dissociation). Glyceraldehyde 3-phosphate dehydrogenase (GAPDH) and U6 were used for normalization. Relative expression (fold change) was calculated using the 2^−ΔΔCt^ method according to a previous study [15]. Each independent experiment was conducted in triplicate. The sequences of the primers used in this study were as follows: PROX1-AS1, forward: 5′-CTAGTTAGCAGGGGCAGCAC-3′ and reverse: 5′-AACAGAGAGGCGTGGAAGAA-3′.

### Transwell assay

The migration and invasion of the HTR-8/SVneo cells were performed using the non-Matrigel and Matrigel Transwell chambers (Merck Millipore, Billerica, MA, USA) as described by Shang et al. [16]. The transfected cells were suspended in a serum-free medium and added to the upper chambers. The complete medium was added to the lower chambers. Then, 24 h post-transfection, the cells in the upper chambers were removed using cotton swabs. The migrated and invaded cells were then fixed with methanol and stained with 0.1% crystal violet. Their numbers were counted using an inverted microscope (Olympus, Tokyo, Japan) in five random fields.

### Flow cytometry

The apoptosis rates of the cells was detected by annexin V-FITC/7-AAD double staining kit (Beyotime, Haimeng, China). The transfected cells were washed with PBS and centrifuged. The concentration of cells was adjusted to 4 × 10^5^ cells/ml. 500 μl binding buffer was added in the cells. Then 5 μl Annexin-V-FITC and 5 μl 7-AAD were added. The cells were incubated in dark for 10 min. Flow cytometry was used to detect apoptosis, excitation wavelength was 488 nm, FITC was detected at 530 nm, PI was detected at 575 nm.

### Dual-luciferase reporter analysis

The Dual-luciferase reporter analysis was performed as reported by Cai et al. [17]. The complementary fragment containing putative binding sites of miR-211-5p (wild-type (WT)-RPOX1-AS1 and WT-caspase-9) and mutant sequences (MUT-RPOX1-AS1 and MUT-caspase-9) were linked to pmir-GLO vectors (Promega). Then, the HTR‐8/SVneo cells were seeded into 24-well plates and co-transfected with WT or MUT vectors and the miR-211-5p mimic and corresponding nc. Lipofectamine 2000 was used for co-transfection. Luciferase activity was measured 48 h after using the Dual-Glo Luciferase Assay System (Promega).

### RNA pull-down assay

The RNA pull-down assay was conducted according a previous study [18]. Biotinylated miR-211-5p (biotin-miR-211-5p) and nc (biotin-nc) were transfected into the HTR‐8/SVneo cells. After 48 h, the transfected cells were lysed for 10 min and harvested. The cells were then incubated with M-280 Streptavidin Magnetic Beads (Invitrogen) at 4°C for 3 h. Later, the cells were washed with pre-cooled lysis. qRT-PCR was performed to measure the enrichment of RPOX1-AS1 and caspase-9.

### Data analysis

Data in this study were analyzed using the GraphPad Prism software v.6.0 (San Diego, CA, USA) and represented as the mean ± standard deviation (SD). Student’s t-test was performed to evaluate the differences between the groups and one-way analysis of variance was used to compare the differences among multiple groups. Statistical significance was set at P < 0.05.

## Results

This study aimed to investigate the effects of PROX1-AS1 on the migration and invasion of placental trophoblast cells. We confirmed that Knockdown of PROX1-AS1 promoted the migration and invasion of HTR-8/SVneo cells via modulating the miR-211-5p/caspase-9 axis.

### Upregulation of PROX1-AS1 in patients with PE

**Figure 1.**
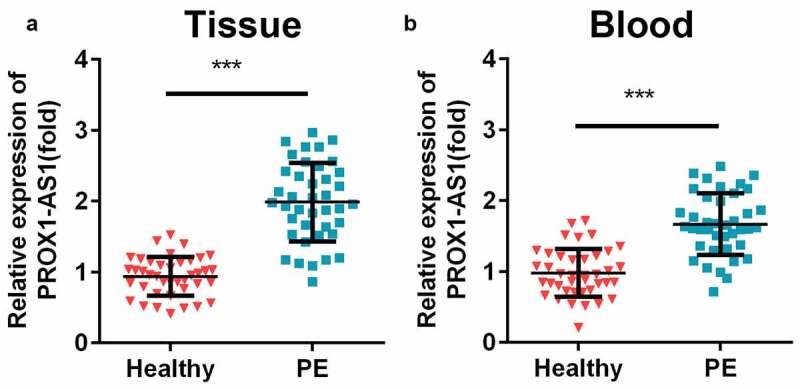
Levels of prospero homeobox 1-antisense RNA 1 (PROX1-AS1) were significantly elevated in the patients with preeclampsia (PE). (a) The expression levels of PROX1-AS1 in the placenta tissues of the patients with PE and the controls were tested using quantitative reverse transcription-polymerase chain reaction (qRT-PCR). (b) The expression levels of PROX1-AS1 in the blood samples of the normal pregnant women and those with PE were detected using qRT-PCR. ***P < 0.001

The expression levels of PROX1-AS1 in the clinical samples were measured using. The results indicated that the expression levels of PROX1-AS1 were significantly increased in the placental samples with PE than the control group (Figure 1(a)). The levels of PROX1-AS1 were also significantly elevated in the blood samples of patients with PE (Figure 1(b)). Additionally, patients with PE were closely associated with short gestational age, high systolic/diastolic blood pressure, proteinuria, and low fetal weight, but not related to the age and body mass index (BMI) of the patients during pregnancy (Table 1).

### Knockdown of PROX1-AS1 promoted the migration and invasion of HTR-8/SVneo cells

**Figure 2.**
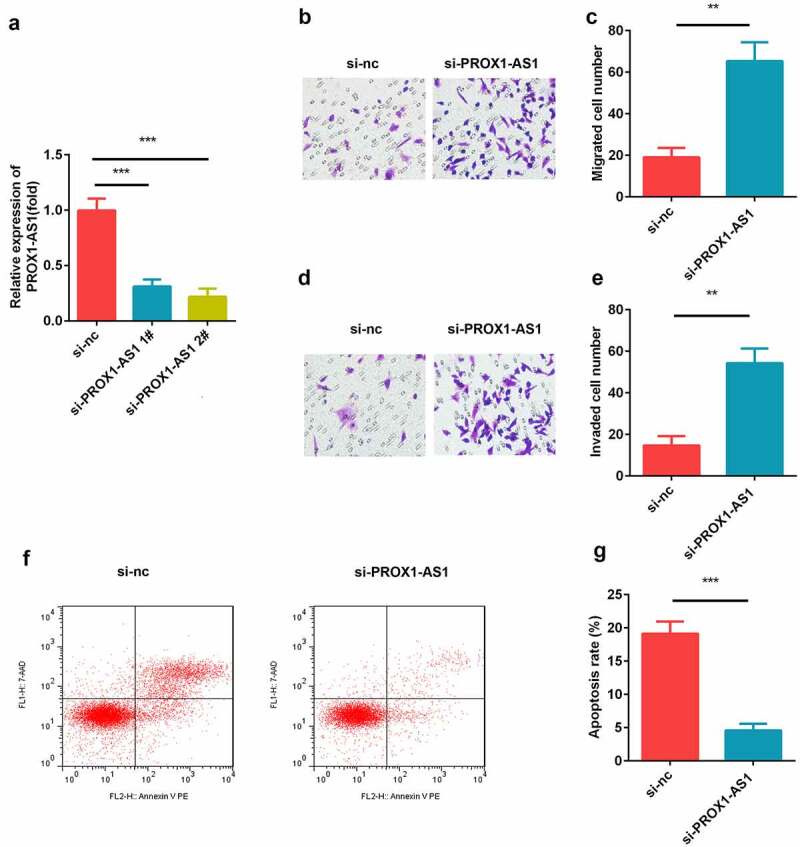
Loss of PROX1-AS1 facilitated the cellular processes of HTR-8/SVneo cells. (a) aRT-PCR was used to measure the transfection efficiency of the transfection of HTR-8/SVneo cells with si-nc, si-PROX1-AS1 1# and si-PROX1-AS1 2#. (b) Cell migration abilities were analyzed by the non-Matrigel transwell assay. (c) Quantification analysis of B. (d) Cell invasion abilities were analyzed by the Matrigel transwell assay. (e) Quantification analysis of D. (f) Flow cytometry was used to measure the apoptosis cells. (g) Quantification analysis of F. ***P < 0.001. **P < 0.01

Trophoblast HTR-8/SVneo cells were transfected with si-nc, si-PROX1-AS1 1#, and si-PROX1-AS1 2#, and the transfection efficiency was tested using qRT-PCR. The data showed that siRNA significantly induced the downregulation of PROX1-AS1, especially si-PROX1-AS1 2#, which was then used for subsequent experiments (Figure 2(a)). Moreover, the knockdown of PROX1-AS1 significantly suppressed the migration of HTR-8/SVneo cells (Figure 2(b,c)). This was consistent with the results of the transwell invasion assay, which revealed that the knockdown of PROX1-AS1 significantly inhibited the invasion abilities of the HTR-8/SVneo cells (Figure 2(d,e)). Additionally, we found that knockdown of PROX1-AS1 significantly suppressed the apoptosis rate of the HTR-8/SVneo cells (Figure 2(f,g)).

### PROX1-AS1 sponged miR-211-5p

**Figure 3.**
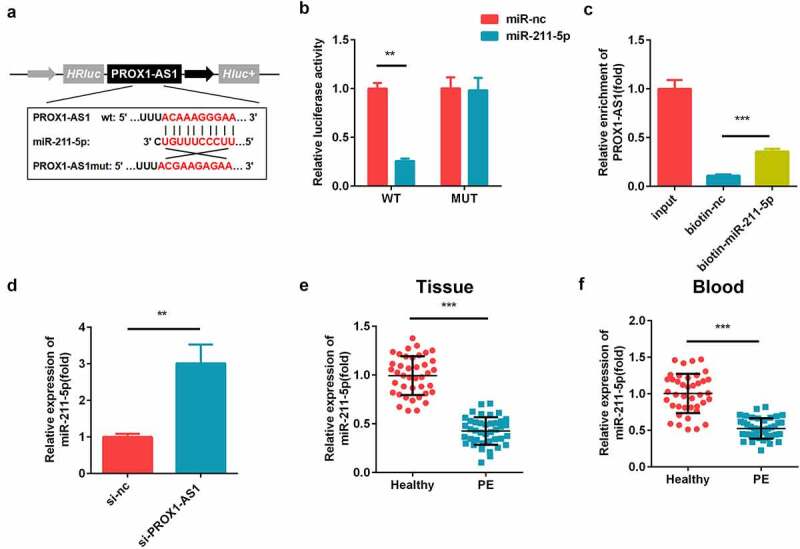
PROX1-AS1 acts as a sponge of microRNA (miR)-211-5p. (a) The complementary binding sites between the 3′-untranslated regions (UTRs) of PROX1-AS1 and miR-211-5p. (b) The luciferase activity was measured using the dual-luciferase reporter analysis and used to verify the targeted relationship. (c) The interaction between miR-211-5p and PROX1-AS1 was tested via RNA pull-down analysis, which confirmed the targeted relationship between them. (d) The expression levels of miR-211-5p were determined using qRT-PCR. (e) The expression levels of miR-211-5p in the placental specimens of the patients with PE as well as the healthy pregnant women were determined. (f) The expression levels of miR-211-5p in the blood samples from the patients with PE as well as the normal pregnant women were determined. ***P < 0.001. **P < 0.01

The putative targets of PROX1-AS1 were predicted. As illustrated in Figure 3(a), PROX1-AS1 could directly bind to miR-211-5p in the 3′-untranslated region (UTR). The luciferase activity of the PROX1-AS1 WT reporter was significantly reduced by miR-211-5p as compared with miR-nc, while there was no influence of miR-211-5p or miR-nc on its activity (Figure 3(b)). Furthermore, PROX1-AS1 was enriched in the biotinylated miR-211-5p transfected cells (Figure 3(c)). The levels of miR-211-5p were significantly elevated after the knockdown of PROX1-AS1 (Figure 3(d)). Meanwhile, the expression levels of miR-211-5p were decreased in the placental tissues and blood samples of the patients with PE as compared with the healthy controls (Figure 3(e,f)).

### Effects of miR-211-5p on the PROX1-AS1-regulated migration and invasion of cells

**Figure 4.**
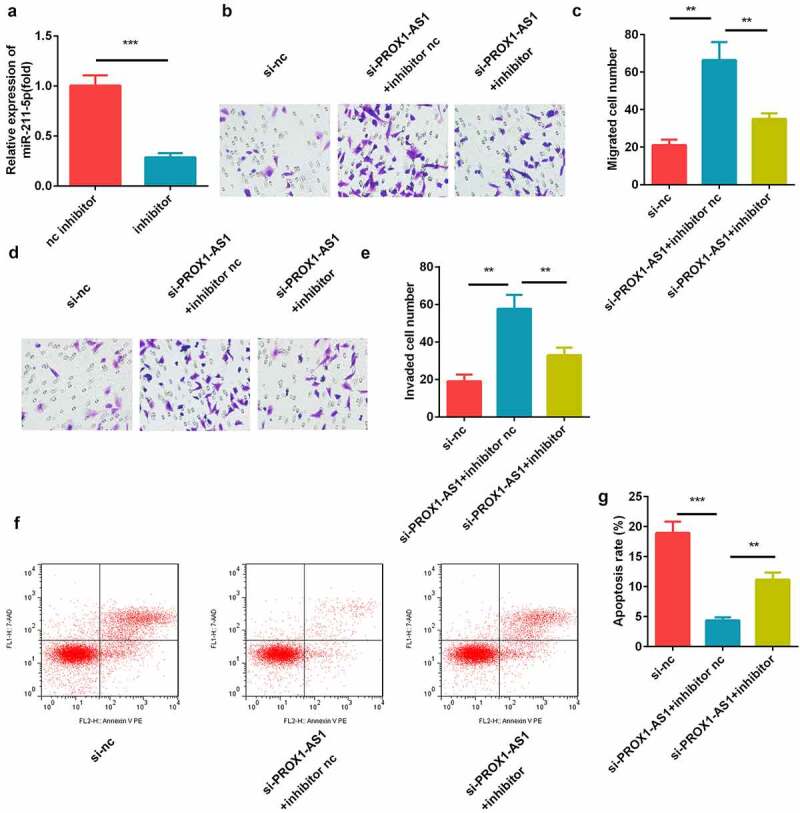
Inhibition of miR-211-5p suppressed the cell migration and invasion induced by the knockdown of PROX1-AS1. (a) qRT-PCR was used to measure the transfection efficiency. (b) Cell migration abilities were analyzed using the non-Matrigel transwell assay. (c) Quantification analysis of B. (d) Cell migration and invasion abilities were analyzed using the Matrigel transwell assay. (e) Quantification analysis of D. (f) Flow cytometry was used to measure the apoptosis cells. (g) Quantification analysis of F. ***P < 0.001. **P < 0.01

The miR-211-5p inhibitor and corresponding nc were transfected into the HTR-8/SVneo cells and the results showed that miR-211-5p was significantly downregulated by the miR-211-5p inhibitor (Figure 4(a)). Moreover, the increase in the migration and invasion abilities of the HTR-8/SVneo cells induced by the knockdown of PROX1-AS1 was remarkably alleviated by the miR-211-5p inhibitor (Figure 4(b–e)). In addition, we confirmed that knockdown of miR-211-5p significantly reversed the effects of si-PROX1-AS1 on the apoptosis rate of the HTR-8/SVneo cells (Figure 4(f,g)).

### miR-211-5p directly targeted caspase-9

**Figure 5.**
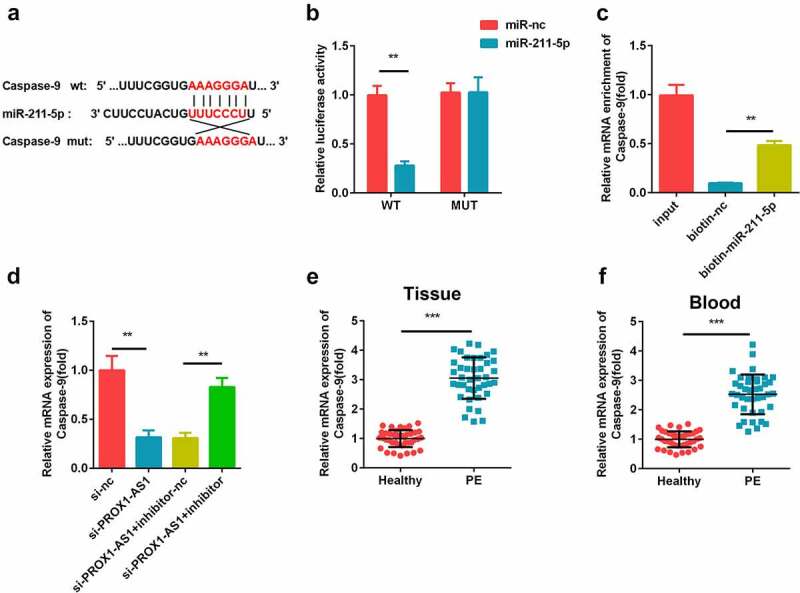
miR-211-5p targeted caspase-9. (a) The complementary binding sites between 3′-UTR of caspase-9 and miR-211-5p. (b) The luciferase assay was used to verify the targeted relationship. (c) The interaction between caspase-9 and miR-211-5p was detected using the RNA pull-down assay. (d) Caspase-9 expression levels were determined using qRT-PCR. (e) Caspase-9 levels were detected in the placental specimens of the patients with PE as well as the healthy pregnant women. (f) Caspase-9 levels were detected in the blood samples of the patients with PE as well as the healthy pregnant women. ***P < 0.001. **P < 0.01

The potential binding sites between miR-211-5p and caspase-9 were predicted using bioinformatics analysis (Figure 5(a)). miR-211-5p reduced the luciferase activity of the caspase-9 WT reporter but not the MUT reporter as compared with miR-nc (Figure 5(b)). Furthermore, caspase-9 levels were enriched in the biotinylated miR-211-5p-transfected cells than the biotin-nc group (Figure 5(c)). Moreover, the expression levels of caspase-9 was decreased by the knockdown of PROX1-AS1, which was reversed by the addition of miR-211-5p (Figure 5(d)). The expression levels of caspase-9 levels were significantly elevated in the placental tissues and blood samples of the patients with PE (Figure 5(e,f)).

### Effects of caspase-9 on the miR-211-5p-mediated migration and invasion of cells

**Figure 6.**
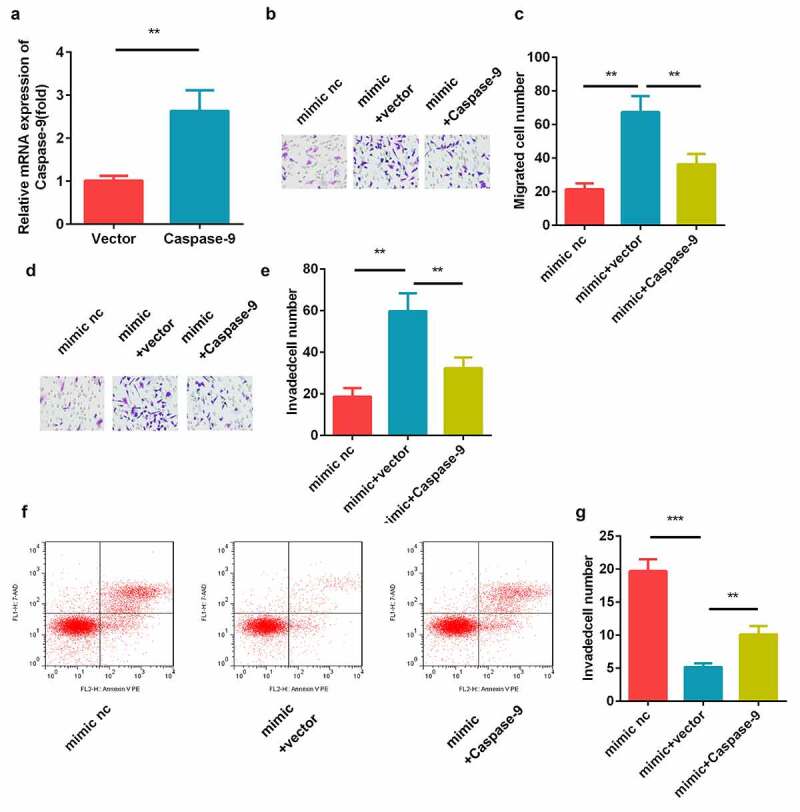
Caspase-9 reversed the cell migration and invasion regulated by miR-211-5p. (a) qRT-PCR was used to measure the transfection efficiency. (b) Cell migration abilities were analyzed using the non-Matrigel transwell assay. (c) Quantification analysis of B. (d) Cell invasion abilities were analyzed using the Matrigel transwell assay. (e) Quantification analysis of D. (f) Flow cytometry was used to measure the apoptosis cells. (g) Quantification analysis of F. **P < 0.01

The results of the transfection efficiency detection using qRT-PCR showed that the caspase-9 levels were elevated in the caspase-9 overexpression plasmid-transfected cells (Figure 6(a)). Moreover, the transwell assay revealed that miR-211-5p facilitated the cell migration and invasion, while caspase-9 inhibited this (Figure 6(b–e)). In addition, we found that caspase-9 overexpression significantly reversed the effects of miR-211-5p mimic on the apoptosis rate of the HTR-8/SVneo cells (Figure 6(f,g)).

## Discussion

Dysfunction of the placenta is closely associated with the pathogenesis of PE [2]. Trophoblast cells are highly invasive endocrine cells that exhibit most of the placental functions [19]. Numerous lncRNAs are involved in human diseases, including PE [20]. Furthermore, the aberrant expression of lncRNAs promotes the incidence and progression of PE by regulating the cellular processes, such as migration and invasion. The migration and invasion of trophoblast cells are critical during the formation of the placenta [21]. Therefore, studying the effects of lncRNAs on the trophoblast cell migration and invasion will aid in understanding the pathogenesis of PE.

The lncRNA PROX1-AS1 acts as a tumor promoter leading to the development of malignant tumors in gastric, papillary thyroid, ovarian, and prostate cancers [22–25]. Additionally, the expression levels of PROX1-AS1 are increased in the placental tissues of the patients with early onset PE [14]. However, the specific function of PROX1-AS1 in the pathogenesis of PE remains unknown. In the present study, we found that PROX1-AS1 was overexpressed in the placental tissues and blood samples of the patients with PE; however, the knockdown of PROX1-AS1 inhibited the apoptosis rate, and promoted the migration and invasion of trophoblast cells. These results indicate that silencing PROX-AS1 might help to alleviate the progression of PE.

miRNAs are small non-coding RNAs that mediate the gene expression by influencing the stability and translation of mRNAs. miR-211 is in 15q13.3, in which the five prime arms of the hairpin are collectively called miR-211-5p. It has been shown that miR-211-5p regulates various biological functions, such as the proliferation, migration, invasion, and apoptosis of cancer cells [26–28]. Moreover, miR-211-5p is also related to chondrocyte differentiation and drug resistance and serves as a prognostic biomarker [29–31]. In PE, the miR-211-5p levels are downregulated [32]. Similarly, in this study, we found that miR-211-5p was downregulated in the placental tissues and blood samples of the patients with PE. Moreover, PROX-AS1 functioned as a competing endogenous RNA (ceRNA) and sponge of miR-211-5p. Additionally, inhibition of miR-211-5p abolished the promotion of cell migration and invasion induced by the knockdown of PROX-AS1. Taken together, our results show that the silencing of PROX-AS1 facilitated the apoptosis, migration and invasion of trophoblast cells by sponging miR-211-5p.

Caspase-9 acts as a key initiator in the intrinsic or mitochondrial pathways. The accumulation of caspase-9 may induce degenerative and developmental diseases, including cancer [33]. Once activated, caspase-9 cleaves and activates caspase-3 and −7, leading to cell apoptosis [34]. Mice lacking caspase-9 are prone to perinatal death due to the inhibition of the apoptosis-induced abnormal brain development [35]. Cas-dependent apoptosis, involving the activation of caspase-3 and caspase-9, is observed in sheep models during the second trimester of pregnancy [36]. Caspase-9 is activated in the placental tissues of the patients with PE and this activation is associated with lipid peroxidation and apoptosis [37]. In the present study, the levels of caspase-9 were found to be increased in the patients with PE, which is consistent with the findings of a previous study [37]. Moreover, caspase-9 reversed the biological functions induced by the overexpression of miR-211-5p. These findings suggest that miR-211-5p inhibits apoptosis, and promotes the migration and invasion of trophoblast cells by targeting caspase-9.

## Conclusion

In conclusion, PROX1-AS1 and caspase-9 are upregulated, while miR-211-5p is downregulated in the placental tissues and blood samples of the patients with PE. Moreover, the knockdown of PROX1-AS1 promotes the migration and invasion, and inhibits the apoptosis rate of trophoblast cells by regulating the miR-211-5p/caspase-9 axis. Therefore, the PROX1-AS1/miR-211-5p/caspase-9 axis may be studies further to develop novel therapeutic strategies for the treatment of PE.

## Highlights

- PROX1-AS1 was up-regulated in PE
- PROX1-AS1 acted as a sponge for miR-211-5p
- PROX1-AS1 modulated the miR-211-5p/caspase-9 axis
